# A PDB-wide, evolution-based assessment of protein–protein interfaces

**DOI:** 10.1186/s12900-014-0022-0

**Published:** 2014-10-18

**Authors:** Kumaran Baskaran, Jose M Duarte, Nikhil Biyani, Spencer Bliven, Guido Capitani

**Affiliations:** 1 Laboratory of Biomolecular Research, Paul Scherrer Institute, OFLC/110, Villigen PSI 5232, Switzerland; 2 Institute of Molecular Biology and Biophysics, ETH Zürich, Zürich 8093, Switzerland; 3 Bioinformatics and Systems Biology Program, University of California, San Diego, La Jolla 92093, CA, USA; 4 National Center for Biotechnology Information, National Library of Medicine, National Institutes of Health, Bethesda 20894, MD, USA

**Keywords:** Protein–protein interfaces, Biological interfaces, Crystal contacts, EPPIC, PISA, PDB

## Abstract

**Background:**

Thanks to the growth in sequence and structure databases, more than 50 million sequences are now available in UniProt and 100,000 structures in the PDB. Rich information about protein–protein interfaces can be obtained by a comprehensive study of protein contacts in the PDB, their sequence conservation and geometric features.

**Results:**

An automated computational pipeline was developed to run our Evolutionary Protein–Protein Interface Classifier (EPPIC) software on the entire PDB and store the results in a relational database, currently containing > 800,000 interfaces. This allows the analysis of interface data on a PDB-wide scale. Two large benchmark datasets of biological interfaces and crystal contacts, each containing about 3000 entries, were automatically generated based on criteria thought to be strong indicators of interface type. The BioMany set of biological interfaces includes NMR dimers solved as crystal structures and interfaces that are preserved across diverse crystal forms, as catalogued by the Protein Common Interface Database (ProtCID) from Xu and Dunbrack. The second dataset, XtalMany, is derived from interfaces that would lead to infinite assemblies and are therefore crystal contacts. BioMany and XtalMany were used to benchmark the EPPIC approach. The performance of EPPIC was also compared to classifications from the Protein Interfaces, Surfaces, and Assemblies (PISA) program on a PDB-wide scale, finding that the two approaches give the same call in about 88% of PDB interfaces. By comparing our safest predictions to the PDB author annotations, we provide a lower-bound estimate of the error rate of biological unit annotations in the PDB. Additionally, we developed a PyMOL plugin for direct download and easy visualization of EPPIC interfaces for any PDB entry. Both the datasets and the PyMOL plugin are available at http://www.eppic-web.org/ewui/#downloads.

**Conclusions:**

Our computational pipeline allows us to analyze protein–protein contacts and their sequence conservation across the entire PDB. Two new benchmark datasets are provided, which are over an order of magnitude larger than existing manually curated ones. These tools enable the comprehensive study of several aspects of protein–protein contacts in the PDB and represent a basis for future, even larger scale studies of protein–protein interactions.

## Background

The Protein Data Bank (PDB) [1] currently contains more than 100,000 high-resolution structures of macromolecules, with protein structures representing the bulk of the data. The PDB is a rich source of information for studying protein–protein interactions, a central theme in biology; it contains hundreds of thousands of protein–protein contacts, a significant percentage of which are biologically relevant. At the same time, thanks to the rapid growth of protein sequence databases, it is now possible to analyze the sequence conservation of nearly all PDB entries. Combining sequence and structural information, in previous work, we tackled the important problem of protein interface classification in crystal structures[2]-[4]. The EPPIC method attempts to distinguish crystal contacts from biological interfaces by a combination of geometric criteria and increased sequence conservation at biological interfaces. It achieves a classification accuracy of about 90% and was implemented in a robust and efficient software package and server (www.eppic-web.org) [3].

EPPIC uses three scores to classify interfaces as biological or crystal contacts. Each residue from the interface is labeled as core, rim or surface, depending on its buried surface area. Six or more core residues are indicative of biological interfaces (geometry score). The other two scores rely on increased conservation of residues participating in the interface relative to the rest of the protein. A multiple sequence alignment is constructed for each chain in the query from homologs with at least 50% identity, and the variability of each position in the alignment is gauged by its sequence entropy. Interfaces are judged biological if they have either a high ratio of average entropy in core residues to rim residues (core-rim score) or highly differential conservation between the core residues and the rest of the surface (core-surface score). The incredible growth of sequence databases has made our conservation-based approaches feasible: 88.1% of protein chains in the PDB with a UniProt reference now (UniProt version 2014_05) have enough homologs at a 50% identity cutoff for reliable calculation of interface conservation. A consensus of these three scores is used for the final classification of an interface as biological or an experimental artifact.

In the current study, we analyze interfaces across the whole PDB. This provides better statistical robustness for assessing methods to classify interfaces as crystal or biological contacts. At the same time, it allows mining of protein–protein interaction data PDB-wide in order to discover possible interesting features of interfaces. While PISA provides a comprehensive database of interfaces and assemblies (http://www.ebi.ac.uk/msd-srv/prot_int/pistart.html) and Ivan *et al.*[5] have reported a PDB-wide clustering study of protein-ligand interfaces, to our knowledge, this study is the first to provide a PDB-wide, evolution-based analysis of protein-protein interfaces.

## Results and discussion

### PDB-wide EPPIC precalculation interface analysis and classification

The importance of using information from the PDB [1] to study protein–protein interactions was highlighted more than 15 years ago in a paper by J. Janin [6]. At the time of publication of his paper, the PDB contained about 6,500 entries, and the SwissProt and TrEMBL databases (later merged into the UniProt database [7]) contained about 68,500 and 150,000 entries, respectively. Since then, the PDB has grown about 15-fold to more than 100,000 entries (as of May 27, 2014), while the UniProt database (version 2014_05) has reached over 52 million entries with a 200-fold increase. Thus, the average number of putative homologous sequences available per PDB entry has greatly increased.

**Figure 1:**
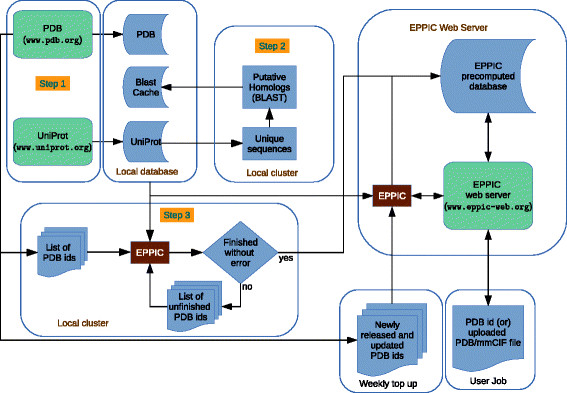
**Schematic representation of the PDB-wide EPPIC precalculation pipeline.** Web servers are denoted by green blocks, local databases and inputs by blue blocks, instances of the EPPIC program by brown blocks.

With the development of the EPPIC software and the availability of sufficient computing power, it is possible to predict the biological relevance of all interfaces in the PDB. An automatic calculation pipeline was implemented to analyze the entire PDB with EPPIC and to store the results in a MySQL database (see Methods for details). Table 1 gives an overview of the database of interfaces. The pipeline, which is shown in Figure 1 as a flowchart, greatly increased the speed, efficiency and usability of the EPPIC web server since all user queries corresponding to existing PDB entries return the precalculated results instead of running the calculation. In this way, the server’s computing power is nearly entirely available for user queries that do not yet correspond to PDB entries. An even more important advantage of our pipeline is the possibility to mine the database and carry out interface analysis on a scale that was previously precluded to our method.

**Table 1:** EPPIC database statistics as of May 27, 2014

Number of entries in PDB database	100147	
Number of entries in EPPIC database ^*a*^	99696	99.54%
Number of protein chains in EPPIC database	129738	
Number of protein chains with UniProt match ^*b*^	124592	96.03%
Number of protein chains having at least 10 homologs with 60% sequence identity	101113	81.16%
Number of protein chains having at least 10 homologs with 50% sequence identity	109761	88.10%
Number of interfaces in EPPIC database ^*c*^	818358	
Number of interfaces classified as bio ^*d*^	114001	13.93%
Number of interfaces classified as xtal	704357	86.07%

In addition to interface predictions, EPPIC also provides convenient information about related sequences for each structure. Easy access to accurate precalculated alignments for all structures in the PDB and to the visualization of sequence entropy on the protein surface could be of use for a variety of tasks and analyses that go beyond interface classification.

**Figure 2:**
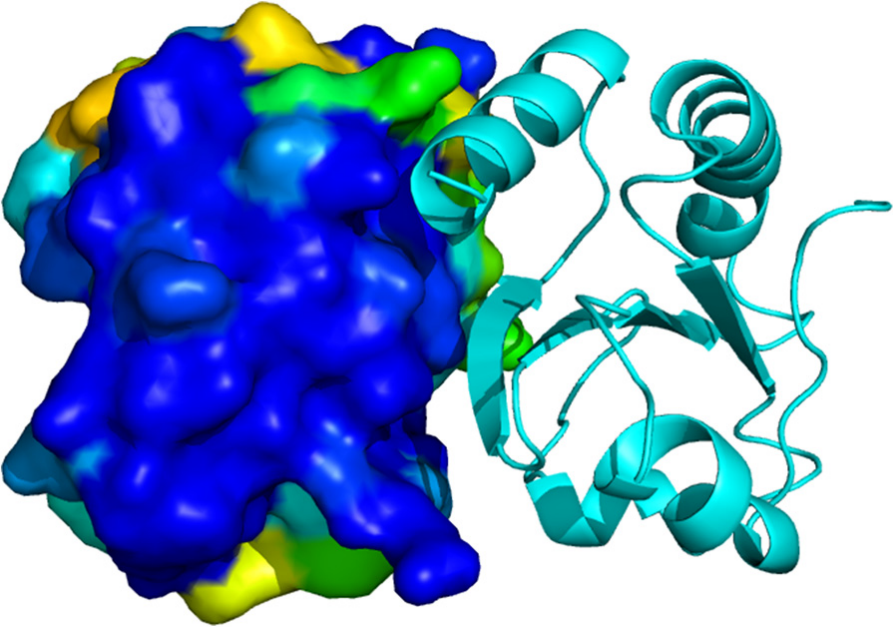
**Example of interface display with the EPPIC Interface Loader plugin for PyMOL.** Interface 1 of entry 2trx (*E.coli* thioredoxin) fetched in PyMOL with the EPPIC Interface Loader plugin and displayed in hybrid mode (surface color-mapped by sequence entropy for one interface partner and cartoon for the other partner).

In addition to the server, we developed a PyMOL plugin that allows users to directly download precalculated EPPIC interfaces into PyMOL (Figure 2). Individual EPPIC interfaces can be requested, or all interfaces for a protein can be loaded simultaneously. Interfaces may be displayed using the default line visualization or in a hybrid mode where one chain is rendered as a cartoon and the other as a surface displaying the sequence entropy for each surface residue.

### Beyond manually curated datasets

One of the major obstacles in developing new methods for crystal interface classification or docking is that of the availability of gold-standard datasets for training and benchmarking where the oligomeric structure has solid experimental backing. This problem has been solved in the past by manual curation. While invaluable as tools for method development and benchmarking, manually curated datasets suffer from several shortcomings: they require large amounts of time to be compiled and validated, they are prone to human errors and, most importantly, they can reasonably cover only a very small percentage of the available structures. The DCbio and DCxtal sets [3], which were used to optimize EPPIC parameters, contain 81 entries each. The Ponstingl dataset [8], which was used in the development of other classification methods [8],[9], contains 86 biological interfaces and 52 crystal contacts. In contrast to those small figures, the number of protein–protein interfaces in the PDB larger than 35 Å ^2^ is on the order of 820,000 as of May 27, 2014.

A benchmark of at least thousands of interfaces would be desirable for the robust training and evaluation of new methods. Rather than attempt to do this manually, the “Many” datasets were assembled automatically based on independent evidence for biological and crystal contacts. This problem was already recognized and partly addressed by Xu and Dunbrack [10], where they provided a dataset of biological interfaces based on their conservation across different crystal forms.

Thus, we based our biological interface dataset (BioMany) on the ProtCID database [10],[11], additionally supplementing it with interfaces validated by NMR. The ProtCID method uses the presence of an interface in multiple crystal forms as an indication that it is biological. The method assumes that crystal contacts are unlikely to be conserved in different lattice forms, while biological interfaces should be strongly bound and consistently present. The ProtCID database [11] clusters similar interfaces based on Pfam domain architecture and structural similarity. Clusters were filtered using conservative thresholds, and then a nonredundant subset of 2,666 interfaces was chosen for inclusion in the BioMany dataset. While most NMR structures are monomers (see Table 2), the remaining oligomers can be confidently assigned as biological interfaces. To avoid systematic biases arising from two different structure determination techniques, the NMR structures were not used directly, but were mapped onto equivalent structures from X-ray crystallography. This resulted in 171 interfaces with six redundant entries, which, along with the ProtCID set, made a dataset with 2,831 unique biological interfaces. Importantly, in the BioMany dataset, we removed interfaces with areas larger than 2,000 Å ^2^ in order to include interfaces with areas belonging only to the difficult-to-classify range [3].

**Table 2:** NMR statistics as of May 27, 2014

**Chains**	**PDBs**	**Percentage**
1	9224	88.80%
2	1021	9.83%
3	54	0.52%
4	65	0.63%
5	9	0.09%
6	3	0.03%
7	2	0.02%
9	1	0.01%
12	7	0.07%
13	1	0.01%

For the set of crystal contacts (XtalMany), we collected homomeric interfaces mediated by screw axes or by pure translations. The concept of interfaces leading to infinite assemblies, used for compiling XtalMany, was first and most elegantly described by Monod [12]. He reasoned that in a hypothetical molecule only two kinds of homomeric interfaces are possible: isologous ones, formed by the same patches in both molecules, and heterologous ones, formed by different surface patches in both molecules. The isologous case necessarily exhibits 2-fold closed symmetry. However, in the heterologous case the monomers may either form a closed ring with rotational symmetry, or they may form a fiber or helix and will continue assembling indefinitely. With the exception of a very small number of fiber-like proteins, infinite assemblies are disadvantageous in nature and can be assumed to be crystal contacts.

In the context of a 3-dimensional crystal, interfaces which are produced by a pure translation or a screw axis can only lead to non-closed assemblies, and can therefore be confidently assumed to be crystal contacts. This is a fact widely acknowledged and used in the literature, although there is no agreement in a single nomenclature for it. Janin [6] uses it to plot the distribution of crystal contacts in known protein crystals (“interfaces having no point-group symmetry”). Krissinel [9] uses it as a fundamental step of his assembly algorithm, any such interfaces of “monomeric units in parallel orientations” are discarded in the enumeration of all possible assemblies compatible with the crystal. Levy, in a review on oligomeric assemblies [13], covers the symmetry topic extensively and discusses the presence of “open symmetries” that can lead to malfunctioning proteins like the case of hemoglobin in sickle cell anemia.

For the XtalMany dataset, interfaces were clustered by sequence and filtered for extremely small interfaces (area<600 Å ^2^), which are very abundant and would be trivial to classify. In addition, entries for which the biological unit annotation in the PDB indicates a helical symmetry were also excluded from the list (only five such structures were found). This resulted in a set of 2,913 crystal interfaces.

**Figure 3:**
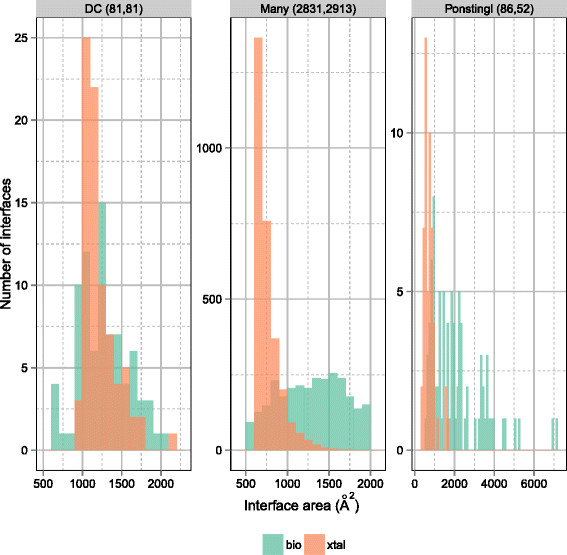
**Interface area distribution of three datasets of interfaces.** The interface areas for crystal contacts (red) and biological interfaces (green) are shown for three interface datasets: DCBio/Xtal (left), Bio/XtalMany (center) and Ponstingl (right). The numbers in parentheses refer to the counts of bio and xtal interfaces in each dataset.

The interface area distribution for the Many benchmarks, as well as the previous DC and Ponstingl benchmarks, are shown in Figure 3. The performance of EPPIC was evaluated on each dataset (Table 3). With the default thresholds, EPPIC obtained a performance in line with that described in the 2012 paper. The performance on the Many datasets is 88% accuracy, 85% sensitivity and 90% specificity, which is comparable to that obtained on the smaller Ponstingl dataset of 91%, 91% and 90%, respectively.

**Table 3:** EPPIC performance based on UniProt 2014_05

**DataSet**	**N(>10 homo.)**	**Method**	**Sensitivity**	**Specificity**	**Accuracy**	**MCC**
DC		Geometry	0.8025	0.7284	0.7654	0.5323
Bio	81(73)	Core Rim	0.8514	0.6462	0.7554	0.5111
Xtal	81(73)	Core Surface	0.8649	0.7059	0.7887	0.5800
		Final	0.9012	0.7160	0.8086	0.6281
Ponstingl		Geometry	0.8721	0.9231	0.8913	0.7789
Bio	86(75)	Core Rim	0.8784	0.7143	0.8333	0.5863
Xtal	52(46)	Core Surface	0.8472	0.8571	0.8500	0.6631
		Final	0.9070	0.9038	0.9058	0.8025
Many		Geometry	0.7549	0.9451	0.8513	0.7143
Bio	2831(2508)	Core Rim	0.8206	0.6580	0.7582	0.4839
Xtal	2913(2368)	Core Surface	0.8900	0.8236	0.8641	0.7142
		Final	0.8531	0.9046	0.8792	0.7590

**Figure 4:**
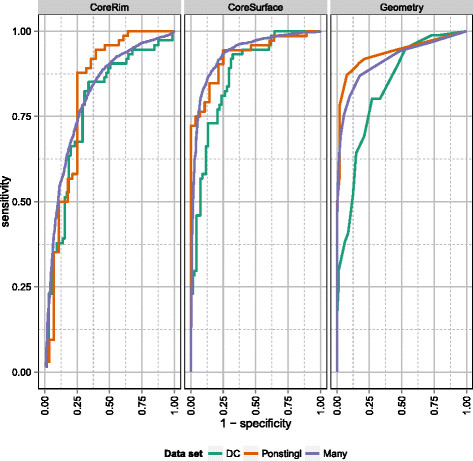
**EPPIC per-indicator performance against three datasets of interfaces.** The ROC curves below show per-indicator EPPIC performance against the same three datasets of interfaces depicted in Figure 3.

Figure 4 provides ROC curves for the three EPPIC indicators (geometry, core-rim, core-surface) versus the three datasets: the core-surface indicator turns out to be consistently the most powerful.

### Revisiting the Janin curve 15 years later

**Figure 5:**
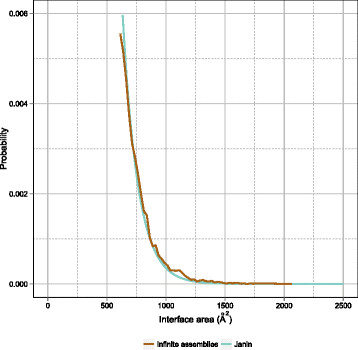
**The Janin curve (1997) revisited.** The Janin curve is plotted against EPPIC calls (based on evolutionary indicators, cyan, and on geometry, green) for all current (May 2014) PDB interfaces larger than 600 Å ^2^ and against all PDB interfaces conducive to infinite assemblies. The curves are plotted as normalized probability versus interface area.

In his landmark paper [6], Janin used a dataset of 1,320 pairwise interfaces derived from 152 crystal forms of monomeric proteins to draw a curve (exponential function) relating the interface area of a lattice contact to the probability of it being a crystal contact. For this fit, Janin used only data points corresponding to contacts with no point group symmetry, which are thus very unlikely to be biologically relevant, as discussed in the previous section (interfaces conducive to infinite assemblies). We set out to compare the Janin curve with our approach, using data from the now 15-fold larger PDB. The result is shown in Figure 5, where the Janin curve appears in light green and the distribution of all interfaces from the current PDB that are conducive to infinite assemblies, encompassing 56,378 interfaces, appears in brown. The two curves overlap very well, testifying to the validity of the original Janin approach and showing that the area distribution of contacts conducive to infinite assemblies has not changed, in spite of the huge increase in the size of the PDB.

### PDB-wide comparison of EPPIC and PISA interface classification

PISA [9] is a well-established method that estimates the thermodynamic stability of an interface to predict whether it should exist in solution (biological interface) or only in the crystalline state (crystal contact). Since PISA makes no use of sequence information, it is completely complementary to our EPPIC method. We carried out a PDB-wide comparison of interface classification by PISA and EPPIC. To obtain the PISA classification for a given interface (biological or crystal), we use the assembly list from the CCP4 [14] command-line PISA application. In a given PISA assembly list, interfaces participating in the assembly are marked as biological and the rest as crystal contacts. There are a few cases in which the interface is classified as “no prediction”; for instance, when PISA gives a “gray” prediction. Out of all the interfaces in the PDB, 96.5% had a valid PISA prediction. Among those, approximately 25% were predicted to be biologically relevant. In comparison, EPPIC predicts 14% of contacts (114,001 of 818,358 contacts) to be biologically relevant. The fact that PISA predicts a larger number of biological interfaces seems to agree with the analysis of Krissinel [15], where, in certain cases, PISA tends to predict too large an assembly due to the binding effect of buffer molecules.

**Figure 6:**
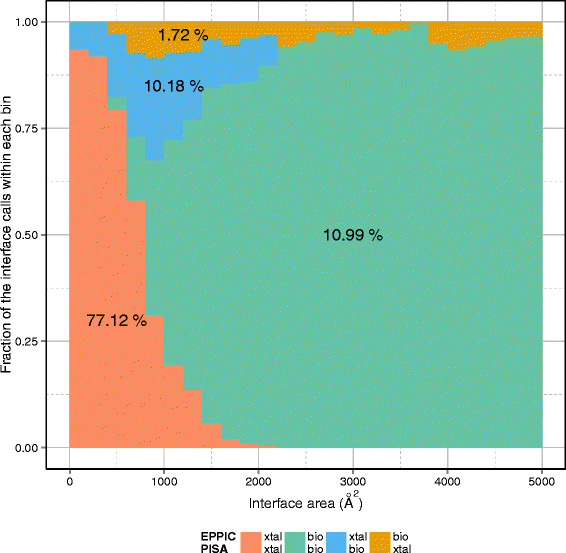
**Interface call comparison between EPPIC and PISA.** The histogram represents the fraction of convergent and divergent interface calls by EPPIC and PISA as a function of interface area. The top call in the color legend corresponds to EPPIC and the bottom one to PISA. The overall percentages of each call combination are also given.

Comparing the results of PISA and EPPIC, we found that the two approaches gave the same call for 88% of interfaces. In Figure 6, we show how the fraction of common calls varies as a function of interface area. Unsurprisingly, the lowest agreement is observed in the 600–1200 Å ^2^ interface area range, where classification is particularly hard. This nicely confirms previous observations based on the analysis of small datasets only [3],[16].

### An estimate of the error rate in PDB author biological unit annotations

An important issue affecting structural bioinformatics analyses is that of errors in the PDB, recognized already in some previous publications [11],[17]-[19]. A very important kind of error is that relating to the biological unit assignment provided in REMARK 350, which is essential for the correct interpretation of a protein’s structure. In many cases, difficulties in determining the correct solution state composition experimentally [20] complicate these assignments. Even in cases where the quaternary structure can be determined experimentally, errors can still be introduced in the annotation process [11].

We attempt an estimation of the error rate in author biological unit annotations in the PDB by comparing them to the most robust EPPIC predictions on a per-interface basis (as opposed to comparing full assemblies). We use the following criteria to keep only the best predictions: first, we consider only crystal structures solved at a resolution better than 2.5 Å and with a refinement free R-factor lower than 0.3; second, we require at least 30 sequence homologs for the EPPIC evolutionary predictions and, third, we require a unanimous call by the three EPPIC criteria (geometry, core-rim, core-surface). Finally, we only retain entries with a core-surface score lower than -3.3 for biological calls and higher than 0.15 for crystal contact calls. These cutoffs ensure that the predictions are only those with the most solid scores and are chosen in such a way that the bio and xtal groups are balanced in number. In the end, this results in 20,000 data points.

**Figure 7:**
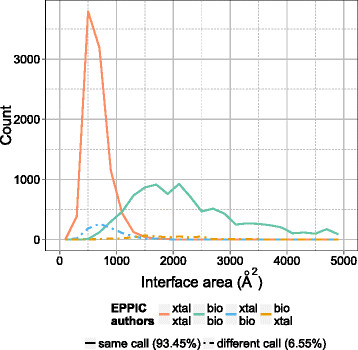
**Author annotation errors in the PDB.** Author annotations are compared to to the EPPIC predictions. The comparison is done on a subset of 10,000 interfaces each from the extrema of the core-surface score distribution. The top call in the color legend corresponds to EPPIC and the bottom one to the author annotation.

Figure 7 depicts an interface call comparison between the most robust EPPIC predictions and the author annotation, similar to the interface call comparison between EPPIC and PISA shown in Figure 6. It is known that a certain rate of error affects author biological unit annotations in the PDB. According to Xu and Dunbrack, it is also not uncommon for an author biological unit annotation not to coincide with the biological unit description of the structure in the corresponding publication [11]. Some previous studies have also attempted to estimate this error rate. In an effort in manual annotation aided by automatic homology-based inference, Levy [19] estimates the error rate to be 14.7%. We estimate the error rate of author annotations at interface level to be 6.6%. Our lower figure indicates a baseline level of the most obvious errors, as we intentionally aimed to find the very clear errors, based on our safest predictions.

### Further interface statistics

**Figure 8:**
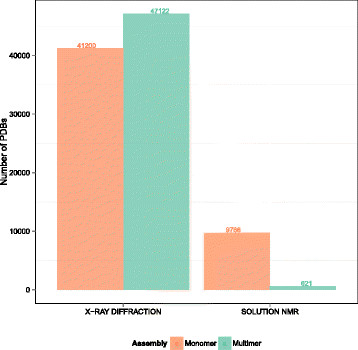
**PDB-wide distribution of EPPIC monomer versus multimer predictions by experimental technique.** PDB entries are considered monomeric (red) if none of their interfaces is classified by EPPIC as bio; otherwise, they are considered multimeric (green).

We additionally analyzed the occurrence of monomers versus multimers in crystal and NMR structures (Figure 8), again using EPPIC classification. An entry was judged as multimeric if it possessed at least one interface classified as biological by EPPIC; otherwise, it was judged as monomeric. According to this approach, X-ray crystal structures are biological multimers in about 53.3% of cases.

**Figure 9:**
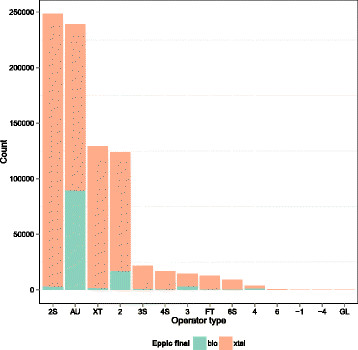
**Interface classification as a function of operator type.** The green portions of the bars represent interfaces classified as bio, the red ones interfaces classified as xtal. Operators are denoted as follows, from left to right: 2S, two-fold screw axis; AU, non-crystallographic symmetry; XT, crystal cell translation; 2, two-fold axis; 3S, three-fold screw axis; 4S, four-fold screw axis; 3, three-fold axis; FT, fractional translation; 6S, six-fold screw axis; 4, four-fold axis; 6, six-fold axis; -1, inversion center; -4, four-fold rotoinversion axis; GL, glide plane.

Since biological multimers can be mediated by non-crystallographic symmetry or by different crystallographic operators, we analyzed the results of interface classification as a function of operator type. The results, depicted in Figure 9, show a difference in the occurrence of biological contacts in the asymmetric unit (i.e. mediated by non-crystallographic symmetry operators), as compared to those via crystal operators. Among the former, more than one-third are biological contacts (37.3%), while contacts through crystal operators were much less likely to be biological. More specifically, 13.4% of the contacts via a pure two-fold crystallographic axis are classified as bio, 19.8% for pure three-folds, 25.2% for pure four-folds and 12.6% for pure six-folds. Only 1% for two-fold screw and three-fold screw axis operators were predicted to be biological, and other types of operators were negligible. The above findings provide information that can usefully be applied in interface classification. The higher percentage of biological contacts mediated by non-crystallographic symmetry may be ascribed to several factors, the most obvious of which are the intrinsic conformational heterogeneity of dimeric assemblies and common practice in the choice of asymmetric unit in PDB entries. In addition, authors may have chosen a lower symmetry space group than allowed by the symmetry of diffraction data, thereby substituting crystallographic operators with non-crystallographic ones. Thus, a dimer mediated by a crystallographic two-fold axis, with a monomer per asymmetric unit, would become a non-crystallographic symmetry dimer.

The role of crystallographic operators for protein crystallization propensity was addressed in an interesting study by Banatao *et al.*[21], analyzing monomeric and dimeric proteins from the PDB. They compared the propensity of dimers and monomers, respectively, to crystallize in space groups containing at least a pure two-fold symmetry axis versus space groups not supporting that symmetry element. They found an enrichment of dimers in the space groups supporting pure two-folds, and they concluded that the fact that homodimers can crystallize both in the asymmetric unit and via a crystallographic two-fold axis provides them with extra crystallization opportunities compared to monomeric proteins. Banatao *et al.* thus advocated the use of synthetic symmetrization for crystallizing recalcitrant monomeric proteins.

## Conclusions

We have carried out the first PDB-wide, evolution-based classification of protein–protein contacts and introduced new criteria to obtain crystal contact benchmark datasets with large numbers (XtalMany). For the BioMany dataset of biological interfaces, we employed multiple crystal form data from the Dunbrack group plus interfaces from structures solved both as NMR dimers and with X-ray diffraction. In this way, we obtained much larger datasets than those that can be reasonably assembled by manual curation. We used the new datasets to benchmark the EPPIC method and confirmed its performance on 5,744 data points. We also revisited, 15 years later, the first approach to interface classification by Janin, who derived a probability curve for an interface to be monomeric based on its buried area. Our data confirm the essential validity of that curve.

We could also provide a lower bound for the estimate of biological unit author annotation errors in the PDB of around 7%. In all likelihood, the actual rate of annotation error is somewhat larger than that. This has been widely acknowledged to be a very important issue that affects any structural biology or bioinformatics analyses. Using computational classification methods prior to other analysis, as a pre-filter, can at least avoid these very clear cases.

Overall, according to the EPPIC classification, if one considers all interfaces in PDB entries, 14% of those are classified as biologically relevant. We further extended our analysis to the relationship between interface classification and other crystallographic aspects, such as crystallographic operator type. Biologically relevant contacts exhibit a skewed distribution as a function of the type of operator: non-crystallographic symmetry is particularly enriched in the fraction of biological contacts it mediates.

## Methods

### PDB-wide EPPIC precalculation pipeline

Figure 1 depicts a flowchart of the EPPIC precomputation pipeline, which involves three major steps. First, the UniProt and the PDB database are downloaded from their respective servers, and a local copy of those databases is created. Second, a list of unique UniProt sequences is prepared and used to perform a BLAST search [22] to find putative homologs for each protein. Sequence search and alignment is the most time-consuming part of the EPPIC calculation, so it is precalculated on an offline cluster, and the results are stored in the local database. The third step is the main EPPIC calculation, which generates predictions for the interfaces of all PDB entries. This is also computed in parallel on a cluster. Its output is then checked for completeness and errors. If hardware or network issues occur, failed jobs are resubmitted to the cluster until full completeness. Finally, the output files are transferred to the EPPIC web server, where the results are stored in a MySQL database. Step 2 takes approximately two days on the local cluster and step 3 requires about five days, assuming an average use of 50 to 60 cores in the cluster (Intel Xeon X5670 with 3 GB RAM each).

The schedule of EPPIC precomputations follows that of the UniProt monthly releases. In addition to that, “top-up” jobs are carried out weekly to include the new releases and updates from the PDB (approximately 200 new entries per week as of the end of 2013). These top-up jobs run on the EPPIC web server every Wednesday morning at 8:00 AM CET, and the results are added to the precomputed database. The server is thus updated just a few hours after the weekly PDB release.

### BioMany and XtalMany datasets

The BioMany dataset is based on the March 20, 2014, update of ProtCID. Clusters were chosen using more stringent criteria than those used in Xu & Dunbrack [10]. Interfaces were required to appear in at least 10 different crystal forms and to be present in at least 80% of the crystal forms known. All entries in the selected interface clusters were then clustered by sequence with BLASTClust [22], using an identity cutoff of 80%. We added additional interfaces to BioMany by using dimeric entries determined by solution NMR. These include a number of small interfaces, which are difficult to detect automatically. Dimeric proteins solved using both solution NMR and X-ray crystallography were identified. The biological interfaces were determined from the NMR structure and subsequently mapped to interfaces from the X-ray structure in order to avoid systematic biases arising from two different structure determination techniques. All structures were required to have a resolution better than 2.5 Å, and a lower area cutoff of 500 Å ^2^ was applied to all interfaces. 171 biological interfaces were found using this procedure, of which six were already present in the selected protein clusters from ProtCID. It is worth noting that the high protein concentration at which NMR structure solution experiments are carried out is likely to stabilize low-affinity interfaces that may exist in a monomer–dimer equilibrium in the cell.

For XtalMany, homomeric interfaces resulting from screw or translational crystal operators were selected. Interfaces were clustered with BLASTClust to 80% sequence identity and filtered for resolution better than 2.5 Å and an area of at least 600 Å ^2^. This resulted in 2,913 interfaces, comparable to the number in BioMany.

### Comparison to PISA

The command-line version of PISA (version 2.0.1) available from the CCP4 package was run for every structure in the PDB and its XML output was parsed. PISA returned a list of one or more possible protein assemblies for each structure. The assemblies in this list were categorized by PISA to be stable, unstable or falling in a gray region of complexation. All unstable assemblies were removed from the list. If the new list contained only gray-region assemblies, we marked all the interfaces in this entry as “no prediction”. Otherwise, we took the largest stable assembly from the list as the final prediction. All interfaces participating in that assembly were tagged as biologically relevant, and the remaining interfaces as crystal contacts. The interfaces were mapped to EPPIC ones by matching the crystallographic operators. If multiple copies of assemblies existed due to multiple copies in the asymmetric unit, we merged those assemblies into a single assembly prediction mapping to EPPIC’s interface clusters. If, in any case, more than one stable assembly with the same size was predicted by PISA, we only took the first one encountered.

## Abbreviations

EPPIC: Evolutionary protein–

protein interface classifier; PDB: Protein data bank

PISA: Protein interfaces, surfaces and assemblies

ProtCID: Protein common interface database

## Competing interests

The authors declare that they have no competing interests.

## Authors’ contributions

JD, KB and GC designed research. All authors analyzed data and wrote the manuscript. All authors read and approved the final manuscript.
